# Stress, inflammation and hippocampal subfields in depression: A 7 Tesla MRI Study

**DOI:** 10.1038/s41398-020-0759-0

**Published:** 2020-02-25

**Authors:** Jonika Tannous, Beata R. Godlewska, Vaishali Tirumalaraju, Jair C. Soares, Phil J. Cowen, Sudhakar Selvaraj

**Affiliations:** 1 Louis A. Faillace, MD, Department of Psychiatry and Behavioral Sciences, University of Texas Health Science Center at Houston, McGovern Medical School, Houston, Texas USA; 2 Department of Psychiatry, University of Oxford, Oxford, OX3 7JX United Kingdom

**Keywords:** Depression, Diagnostic markers

## Abstract

Experiencing stressful events throughout one’s life, particularly childhood trauma, increases the likelihood of being diagnosed with Major Depressive Disorder (MDD). Raised levels of cortisol, and markers of inflammation such as Interleukin (IL-6) and C-reactive protein (CRP), have been linked to both early life stress and MDD. We aimed to explore the biological stress signatures of early stress and MDD on hippocampal sub regional volumes using 7 Tesla MRI imaging. A cohort of 71 MDD patients was compared against 46 age and sex-matched healthy volunteers. MDD subjects had higher averages of IL-6 and CRP levels. These differences were significant for IL-6 levels and trended for CRP. There were no significant group differences in any of the hippocampal subfields or global hippocampal volumes; further, there were no hippocampal subfield differences between MDD subjects with high levels of our biological stress measures and MDDs with normal levels.

## Introduction

Major depressive disorder (MDD) is a disease affecting around 16% of people over the age of 13 in the US^1^. This prevalent mood disorder greatly impacts a patient’s quality of life and is the leading cause of disability around the world^2^. Experiencing stressful events throughout one’s life, particularly childhood trauma, further increases the likelihood of being diagnosed with MDD^3^. These stresses often exacerbate chronic medical issues, such as heart disease, diabetes and other inflammatory disorders^4–6^. To quantify biological stress, studies have used measured levels of cortisol, a stress hormone, as well as Interleukin (IL-6) and C-reactive protein (CRP), two markers of inflammation. Increased levels of all three of these measures have been linked to both early life stress and MDD^7–9^.

Psychological stress and MDD have also been reported to affect brain anatomy, with numerous brain regions shown to have decreased volumes in patients^10^. Indeed, the hippocampus, a subcortical structure critically involved in memory and cognition, has been shown to be sensitive to stress and smaller hippocampal volumes are characteristic of various mood disorders, including MDD^11–13^. Although global hippocampal volume may differ between populations, the hippocampus is in fact made up of anatomically and functionally distinct subfields that may be differentially sensitive to stressors and play varying roles in mood disorder pathophysiology^14^. Therefore, investigating whether specific subfields are linked to MDD may serve to provide greater clarity as to the mechanisms behind the disease^15^. There have been several studies published on hippocampal subfields in MDD, some of which have reported volume decreases in the cornu ammonis (CA) and dentate gyrus, while others have found no significant differences between MDD subjects and healthy controls (HCs)^16–18^.

More recent studies have conducted their analyses using the automated segmentation algorithm released in Freesufer 6.0. This algorithm was developed using an atlas based on ex vivo brain tissue, which was scanned under ultra-high field strength. It has been shown to result in more accurate segmentation of the hippocampus, and it is able to label the molecular layer (ML) and granule cell layer (GCL), both which were not available in previous segmentation algorithms^19^. Yet, there are relatively few studies published that use the novel algorithm, and conflicting results as to whether differences between the subfields of MDDs and HCs are significant^20–23^. Only one of these studies used high-field 7T scanners to acquire their hippocampal neuroimaging data. Due to its complex structure and distinct subfields, the hippocampus can particularly benefit from the enhanced resolution and contrast acquired by a 7T scan. Indeed, a study comparing 7T images with ex vivo anatomy found 7T images to provide excellent anatomic detail of the hippocampus^24^.

Recent developments in ultra-high-field (UHF ≥ 7T) imaging allow for enhanced exploration of both structure and function of the brain. Due to increased signal-to-noise (SNR), spatial resolution, and contrast-to-noise (CNR) ratios, finer details and difficult to scan structures can be better visualised^25^. UHF imaging simultaneously poses a number of challenges, such as non-uniform radiofrequency fields, enhanced susceptibility artifacts and higher radiofrequency energy deposition in the tissue creating a higher likelihood of focal RF heating and tissue damage. These issues, however, can be solved at the level of hardware (such as coils), software (such as using particular scanning parameters) and post-scanning data processing (such as bias correction)^26^. UHF imaging may also induce transient physiological effects such as nausea, dizziness, metallic taste, magnetophosphenes and increased blood pressure in some subjects. However, these effects are temporary, pose minimal risk to the subject, and do not create major obstacles to scanning^26^. Therefore, given the great potential for more accurate and reliable hippocampal segmentations, using 7T imaging data to study the hippocampal subfields of MDD patients may provide more definitive conclusions about the neurobiology of the hippocampus in MDD.

This study aimed to explore the brain anatomical and biological stress signatures of MDD and how early exposure to psychological trauma may affect the presentation of the illness using 7T MRI scans. We hypothesised that our MDD subjects would have higher levels of biological stress measures, specifically cortisol, IL-6 and CRP. Despite the hippocampal subfield literature being mixed, we hypothesised that we would find smaller global hippocampal volumes and reductions in the dentate gyrus and cornu ammonis in the MDD group.

## Methods

### Subjects

Adult volunteers between the ages of 18 and 65 were recruited for this study. Patients meeting DSM-IV diagnosis of MDD without other Axis 1 conditions, such as bipolar disorder, psychosis, or substance dependence (determined using the Standard Clinical Interview for Diagnostic and Statistical Manual for Mental Health Disorders—Fourth Edition)^27^ were included. Patients with a clinically significant risk of suicidal behaviour, or a need for urgent drug treatment were excluded. Healthy controls without a current or past history of Axis I disorders on DSM-IV were included. Any potential subject with contraindications to magnetic resonance (MR) imaging, who was pregnant or breastfeeding, or was involved in a research project during the month preceding the study was excluded from the study sample as well.

Mood ratings were measured using the Hamilton Rating Scale for Depression (HAM-D)^28^ and the Beck Depression Inventory (BDI)^29^, while anxiety ratings were scored using the Spielberger State Anxiety Inventory (STAI)^30^. We also measured anhedonia with the Snaith–Hamilton Pleasure Scale (SHAPS)^31^ and fatigue with the Chalder Fatigue Scale (CFS)^31,32^. Collectively, these scores were used to capture illness severity of a given subject. Childhood trauma was measured using the Childhood Trauma Questionnaire (CTQ)^33^. All subjects included in this study gave informed written consent, and this study was approved by the National Research Ethics Service Committee (NRES), South-Central Oxford B.

### Biological stress measures

Venous blood samples were taken at the time of scanning. Five millilitres were assayed on the same day for high sensitivity C-Reactive Protein (hsCRP), using a standard immunoturbidimetric method on an Abbott c 16 000 automatic chemistry analyzer (Abbott Diagnostics, Maidenhead, UK). Another 5 mL were collected into an EDTA tube, centrifuged for 10 min at 1250*×g* within 30 min to separate plasma, which was then stored at −30 °C until assay. On study completion, the plasma samples were transferred in a single batch, in unlinked anonymized form, for Human IL-6 Immunoassay using a Quantikine® HS (high sensitivity) ELISA kit (R&D Systems, Abingdon, UK). The assay was performed in duplicates.

Salivary cortisol was assayed in duplicate using a commercial, ELISA-based method according to manufacturer’s instructions (Salimetrics).

### Imaging

**Fig. 1:**
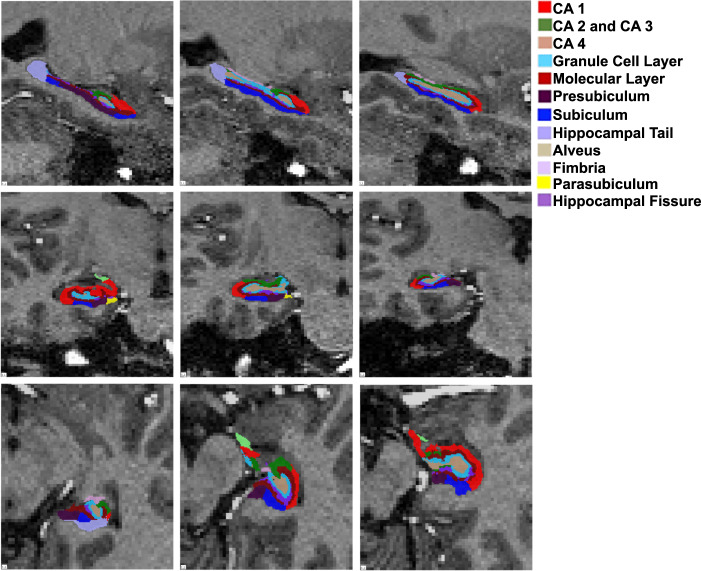
A sample segmentation of the left hippocampal subfields of a healthy subject. CA cornu ammonis.

All participants were scanned at the Functional Magnetic Resonance Imaging of the Brain (FMRIB) Centre in Oxford using a 7T Siemens MAGNETOM scanner (Siemens, Erlangen, Germany) with a Nova Medical 32 channel receive array head coil. The whole-brain 1 mm isotropic T1-MPRAGE image was obtained (MPRAGE: repetition time = 8.60 ms, echo time = 4.00 ms, flip angle = 7°, field of view = 192 mm, GRAPPA factor = 4). All MRI scans were visually inspected for anatomical abnormalities before being processed. Due to greater intensity inhomogeneity in 7T scans compared to 3T, images were bias corrected in SPM12 before processing as per the Freesurfer recommendations, with FWHM set at 18 mm, sampling distance at 2 mm, and bias regularisation at 0.001 (https://surfer.nmr.mgh.harvard.edu/fswiki/). All other parameters remained at default. Freesurfer 6.0 was then used for motion correction, intensity normalisation, automated topology corrections and fully automatic segmentation of cortical and subcortical regions^34–36^. Segmentation of the hippocampal subfields was conducted using the automated segmentation method developed by Iglesias et al, which, as shown in Fig. 1, results in the parcellation of 12 hippocampal subfields^19^. All segmentations were visually assessed by taking the segmented image and superimposing it on the corresponding structural T1-weighted image. Only one subject was excluded from analysis due to poor subfield segmentation.

In order to optimise the power of our study, we selected eight hippocampal subfields to include in our analysis based on prior findings in the literature^20,37,38^. The following subfields were included in our analysis: CA1, CA2 and CA3 (noted as CA3 due to the indistinguishable MR contrast between CA2 and CA3), CA4, Granule cell layer, molecular layer, presubiculum, subiculum and the hippocampal tail.

### Statistical analysis

All statistics were performed in RStudio 3.5.0. Chi-square and independent, two-sample *t*-tests, and Mann–Whitney tests were used to test for demographic differences between groups. Due to the visibly skewed nature of the mood scores and biological stress measure data, shapiro tests of normality were used to evaluate whether parametric testing would be appropriate to evaluate group differences. Mann–Whitney tests were used in lieu of independent two-sample *t*-tests if the measures were not normally distributed. As an exploratory investigation into biological stress, for each of the biological stress measures, we used the average level across all subjects to divide MDD subjects into ‘low’ and ‘high’ level groups and tested whether the groups differed in subfield volume, CTQ scores, illness duration, and illness severity using Mann–Whitney tests.

General linear models were conducted to assess the effect of diagnostic group on hippocampal subfield volumes. We also tested whether MDD subjects in the ‘high’ biological stress measure groups had different subfield volumes than HCs. In order to fully explore gender effects, we also divided the data into male and female datasets and repeated the general linear model analysis to investigate hippocampal subfield differences between MDDs and HCs. In addition, partial correlations were used to examine the relationship between subfield volumes, biological stress markers, mood scores, and illness duration for MDD subjects. FDR corrections were used to adjust for multiple comparisons for all the general linear models and partial correlations. General linear models and partial correlations also controlled for age, gender and total intracranial volume. Significance threshold was set at *p* < 0.05.

## Results

### Biological stress measures

Demographic, mood, and biological stress data are summarised in Table 1. There were significant group differences in IL-6 levels, while CRP differences trended towards significance (*W* = 770.5, *p* = 0.02; W = 829.5, *p* = 0.08). There was a positive correlation between IL-6 and CRP levels. Cortisol, however, did not differ significantly between groups and did not have a significant relationship with the other stress measures collected. In addition, there were no significant relationships between any biological stress measures and CTQ or illness duration. We used the average level of each of the biological stress markers to subdivide the MDD subjects into ‘low’ and ‘high’ groups; these averages were: cortisol, 0.209 μg/dL; IL-6, 1.27 pg/mL; CRP, 0.88 mg/L. Mann–Whitney tests analysing hippocampal subfield differences between “low” and ‘high’ groups were not significant for any of the biological stress measures. We also tested for differences in illness duration and mood rating scale scores. There was a trend that indicated subjects with higher CRP scores having higher SHAS scores (low: *m* = 32.69 ± 5.98; high: *m* = 35.94 ± 7.84; *W* = 243.5, *p* = 0.066). No other differences were significant or approached significance.

**Table 1:** Demographic information of study participants.

	HC (46)	MDD (71)	*t*		*W*	*X*^2^	*p*
Age (years)	31.5 ± 10.5	31.6 ± 10.2	0.07				0.94
Gender						<0.001	1.00
Male	45.7% (21)	45.1% (32)					
Female	54.3% (25)	54.9% (39)					
Cortisol (μg/dL)	0.2 ± 0.2	0.2 ± 0.1		1118.5			0.89
CRP (mg/L)	0.5 ± 0.5	1.1 ± 1.6		829.5			0.08
IL-6 (pg/mL)	1.2 ± 1.3	1.4 ± 1.1		770.5			0.02
CTQ							
Emotional abuse	6.6 ± 2.4	12.6 ± 5.7		455.5			<0.001
Physical abuse	5.3 ± 1.0	7.7 ± 3.8		781.5			<0.001
Sexual abuse	5.2 ± 0.7	6.6 ± 3.9		1127			<0.001
Emotional neglect	8.1 ± 3.5	14.1 ± 5.0		501.5			<0.001
Physical neglect	6.0 ± 2.1	8.5 ± 3.8		781.5			<0.001
Denial	0.5 ± 1.0	0.2 ± 0.6		1662.5			0.07
Currently on medication	–	22.5% (16)					
Illness duration (years)	–	10.9 ± 11.0					
BDI	2.1 ± 4.7	30.8 ± 8.6		26.5			<0.001
HAM-D	0.6 ± 2.4	22.1 ± 5.5		10			<0.001
STAI	26.4 ± 7.1	52.9 ± 12.0		100			<0.001
CFS	10.1 ± 3.2	23.2 ± 4.8		23			<0.001
SHAS	19.2 ± 5.5	33.4 ± 6.6		165.5			<0.001

*HC* healthy controls, *MDD* major depressive disorder, *BDI* beck depression inventory, *HAM-D* Hamilton Rating Scale for depression, *STAI* Spielberger state anxiety inventory, *CFS* Chalder Fatigue Scale, *SHAPS* Snaith–Hamilton Pleasure Scale.

### Hippocampal subfields

**Fig. 2:**
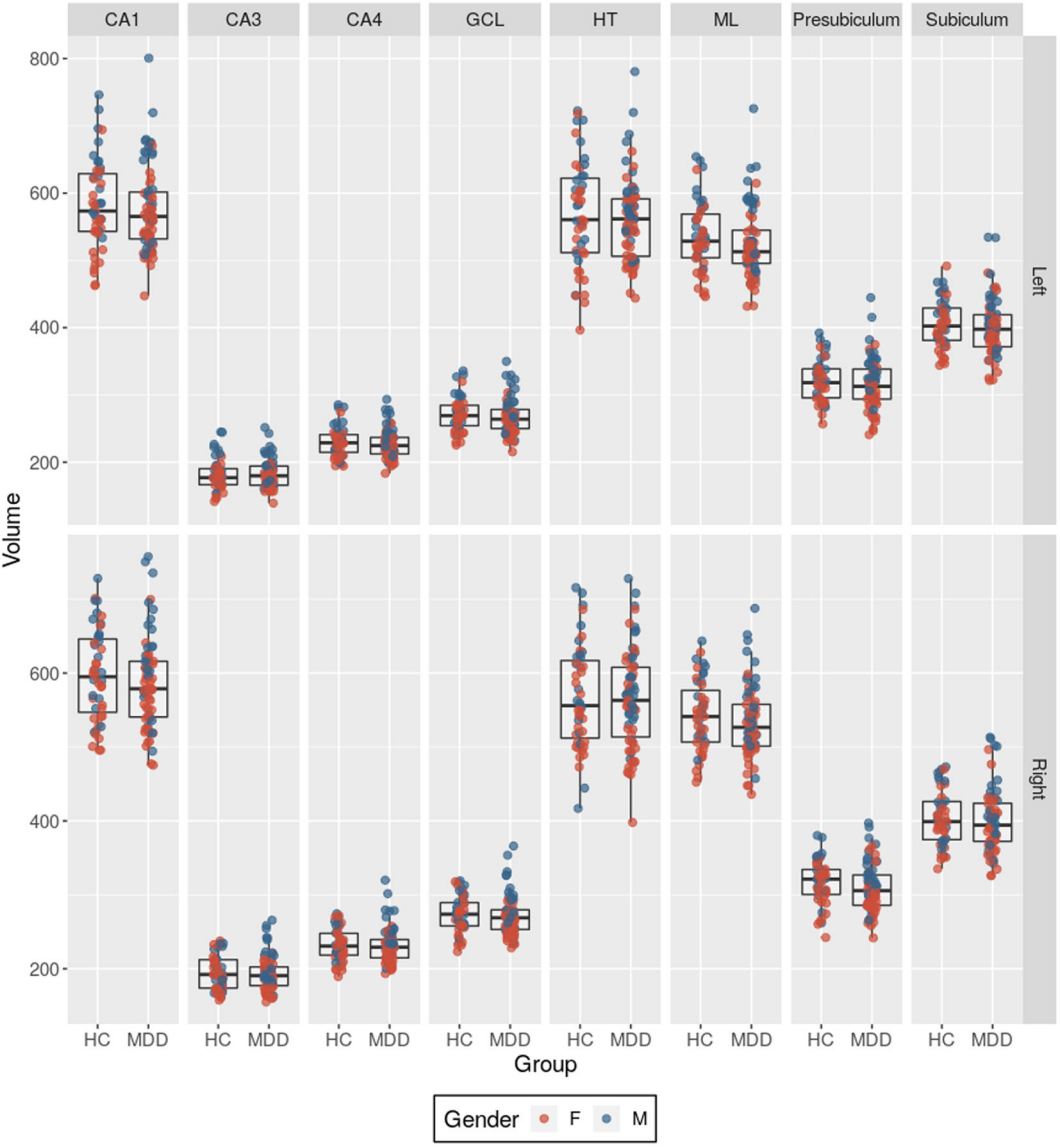
Hippocampal subfield volumes of all subjects (mm^3^). Subfield volumes MDDs and HCs were compared using general linear models that were then FDR corrected for multiple comparisons. No subfield differences were found between subject groups. HC Health Controls, MDD Major Depressive disorder, F Female, M Male, CA Cornu Ammonis, GCL Granule Cell Layer, HT Hippocampal Tail, ML Molecular Layer.

There were no significant group differences in any of the hippocampal subfields examined (Fig. 2). Global hippocampal volume differences were also not significantly different. Although we found IL-6 levels to increase along with right CA3 volumes (*t* = 2.751, *p* = 0.007), this did not survive multiple comparison correction. There were also no hippocampal subfield differences between MDD subjects with high levels of our biological stress measures and MDDs with normal levels. There were no significant relationships between any subfield and any other biological stress measure, CTQ score, illness duration, or mood rating scale. We separated males and females and repeated the hippocampal subfield analysis on both datasets and found no differences between MDDs and HCs. We also compared the hippocampal subfield volumes of our MDD subjects who had high cortisol, IL-6, and CRP levels relative to the rest of the study sample to our HC group. We found the right CA 1 to be smaller in high cortisol MDD patients compared to controls [MDD high cortisol- M = 567.6 ± 47.2 mm^3^, HC- *M* = 597.4 ± 61.9 mm^3^, *F*(1,66) = 5.0, *p* = 0.03]. This, however, did not survive FDR correction for multiple comparisons across subfields. The hippocampal subfield volumes of MDD subjects who had high levels of IL-6 and CRP did not differ significantly than those of HCs.

## Discussion

Our study sought to investigate how stress and trauma affect the pathophysiology and symptomatology of MDD. Our MDD cohort had higher levels of trauma than our HC’s in all CTQ domains except for denial. Furthermore, MDD subjects were shown to have higher levels of IL-6 and CRP, two common markers of biological stress that we also found to be correlated with one another. Our biological stress measures did not have significant relationships with childhood trauma and did not appear to be related to hippocampal subfield volumes. Although we expected to find hippocampal subfield volume differences between MDD and HC subjects and those with low versus high CTQ scores, our data did not show these trends. As aforementioned, due to the lack of consistent findings regarding hippocampal subfields in MDD, more studies are needed for a clearer characterisation of the role of the hippocampus in the pathophysiology of MDD^20–23^.

In addition, these discrepancies in the literature may be due to the heterogeneity of inherent to mood disorders, and future work emphasising computational approaches to classifying subjects has the potential to better explore MDD and other mood disorders^39^. Additionally, a power analysis based on the effect size of 0.35 reported in a meta-analysis on the hippocampus in MDD indicated that our study had a power statistic of 0.38^11^. Regarding the association between CTQ scores and hippocampal volumes, studies have reported subjects with higher scores having smaller global hippocampal volume as well as deficits in certain subfields, specifically the CA3, subiculum, and the dentate gyrus^40,41^. However, a meta-analysis examining the effects of childhood maltreatment on global hippocampal volume found an effect size of just 0.08^42^. Such a small effect size, in turn, leaves our study with a power statistic equal to 0.07. Therefore, despite having a relatively large sample when compared to published hippocampal subfield studies, the power of our analysis remains limited due to the small effect sizes characteristic of hippocampal abnormalities. Future studies with even larger cohorts and multi-site datasets will have greater power and may provide a clearer picture on the role of hippocampal subfields in MDD.

Our findings did, however, imply greater illness severity in MDD subjects with CRP levels above the calculated threshold compared to MDDs in the lower range. As this pattern trended, a more balanced sample of MDD subjects with and without high CRP levels will serve to further elucidate the role of CRP in MDD symptomology. Moreover, when examining the distribution of CRP and IL-6 levels in our sample, we noted that our subjects had lower levels than those reported in studies showing associations between these two biological stress measures and grey matter volumes, which may also factor into our null findings^43,44^.

Ultimately our study reports on the intersection between trauma, stress, and hippocampal structure. Due to the rarity of our imaging data being acquired using high-field 7T scanner and the relatively large size sample, we believe that our lack of significant findings regarding the impact that childhood trauma and MDD have on hippocampal subfields remains a useful contribution to a mixed body of literature in need of further investigation.
